# Supplementary material for: Automation and improvement of WBC mechanical profiling in deformability cytometry
Source: Biophys J. 2025 Oct 10;125(12):3048–59. doi: 10.1016/j.bpj.2025.10.007 (PMC13507180; doi:10.1016/j.bpj.2025.10.007)
Supplement: Supplementary file 1 [file mmc1.pdf]

Biophysical Journal, Volume *125*

Supplemental information

Automation and improvement of WBC mechanical profiling in deformability cytometry

Sara Kaliman, Shada Abuhattum, Benedikt Hartmann, and Jochen Guck

Supplemental Information

Automation and Improvement of WBC Mechanical Profiling in Deformability Cytometry

Sara Kaliman,[1,2,*] Shada Abuhattum,[1,2] Benedikt Hartmann,[1,2,3] and Jochen Guck[1,2,3,†]

[1]*Max Planck Institute for the Science of Light, 91058 Erlangen, Germany*

[2]*Max-Planck-Zentrum für Physik und Medizin, 91058 Erlangen, Germany*

[3]*Friedrich-Alexander-Universität Erlangen-Nürnberg (FAU), 91054 Erlangen, Germany*

[*]sara.kaliman@mpl.mpg.de

[†]jochen.guck@mpl.mpg.de

I. SUPPLEMENTARY FIGURES

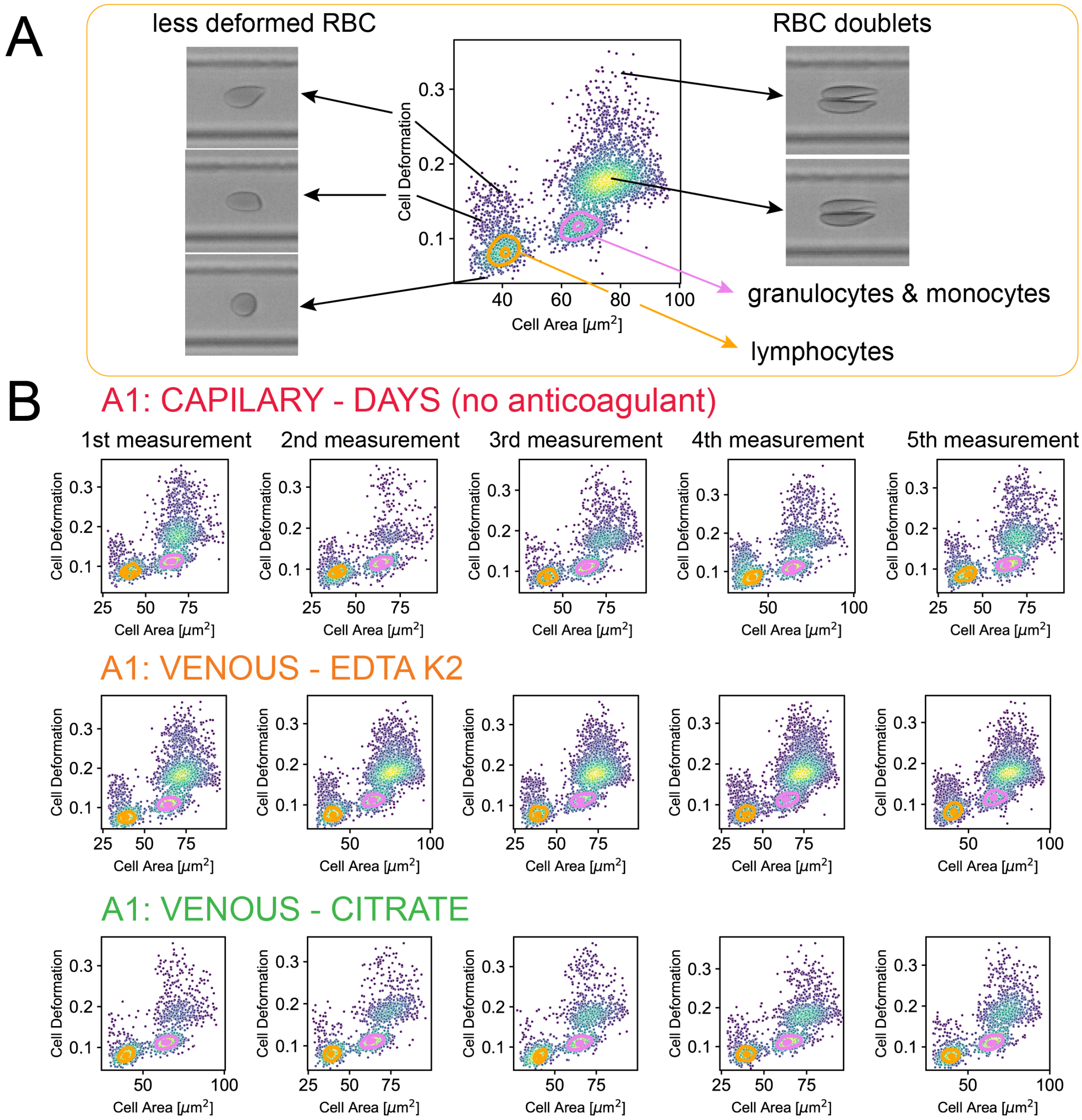

FIG. S1: Kernel density estimate (KDE) plot of target and non-target cell populations within standard WBC box-filters for area and deformation, and with porosity threshold of 10%. (A) Lymphocyte regions are predominantly surrounded by less deformed RBCs, whereas granulocytes and monocytes cluster is bordered by RBC doublets. (B) Comparison of non-target population abundance across three experimental sets, each comprising five measurements: A1: capillary-days (no anticoagulant was used), A1: venous-EDTA and A1: venous-citrate. All blood samples are collected from the same donor (A1) and KDE two levels indicate 50% and 95% of distribution density. Notably, using citrate results in fewer RBC doublets compared to using EDTA K2.

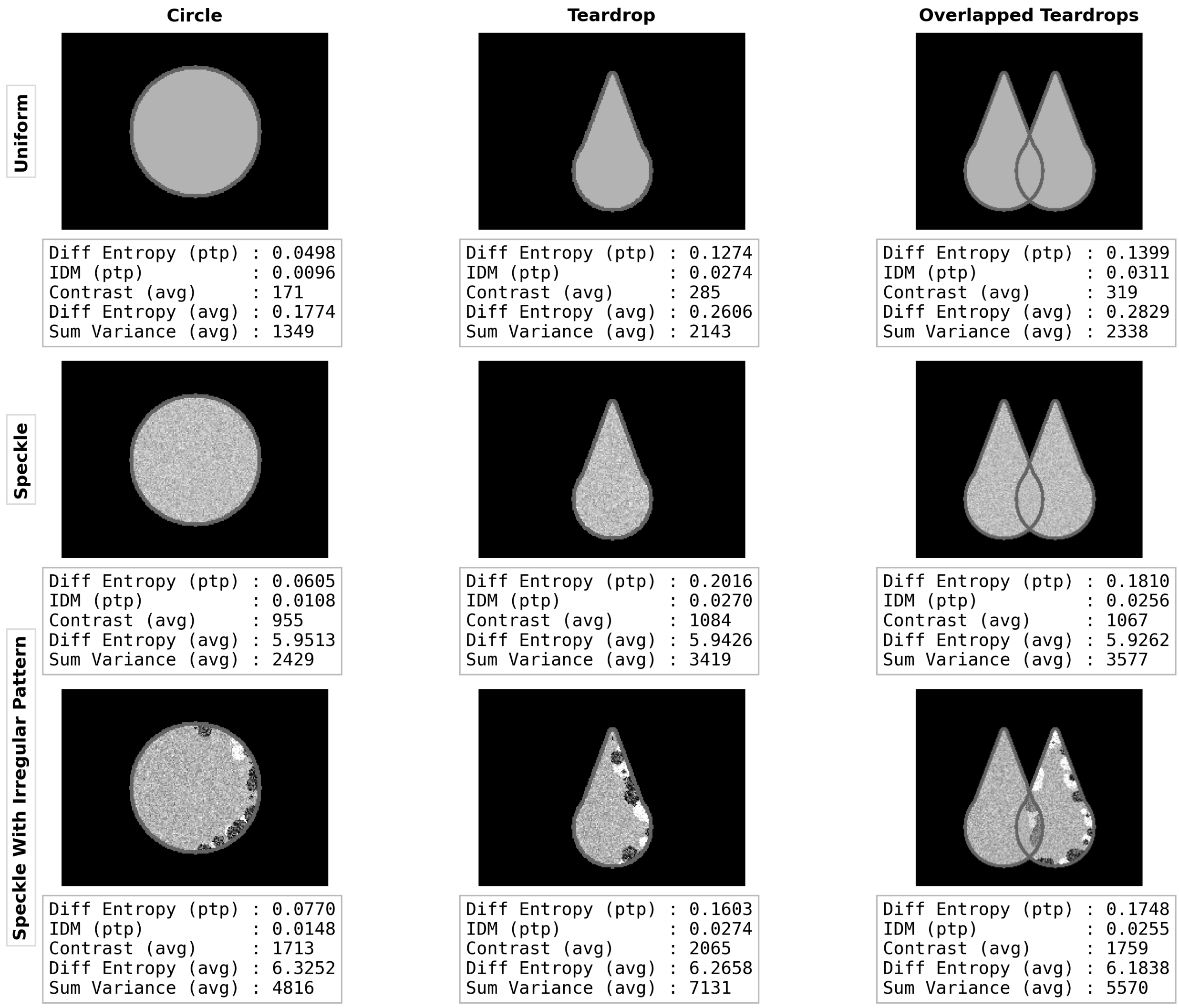

FIG. S2: Visual guide illustrating the five Haralick features used in automated WBC classification. Columns present synthetic shapes: circle, teardrop, overlapping teardrops, while rows depict different textures: uniform, speckle, and speckle with irregular patterns. These schematic renderings mimic the diversity of shapes and textures observed in cells captured by the initial box filters, including RBC doublets and WBCs. Among WBCs, some exhibit more regular speckled textures (e.g., lymphocytes and monocytes), while others display more irregular textures (e.g., eosinophils). For each image, the accompanying table reports values of: Difference Entropy (ptp), Inverse Difference Moment (IDM) (ptp), Contrast (avg), Difference Entropy (avg), and Sum Variance (avg)

.

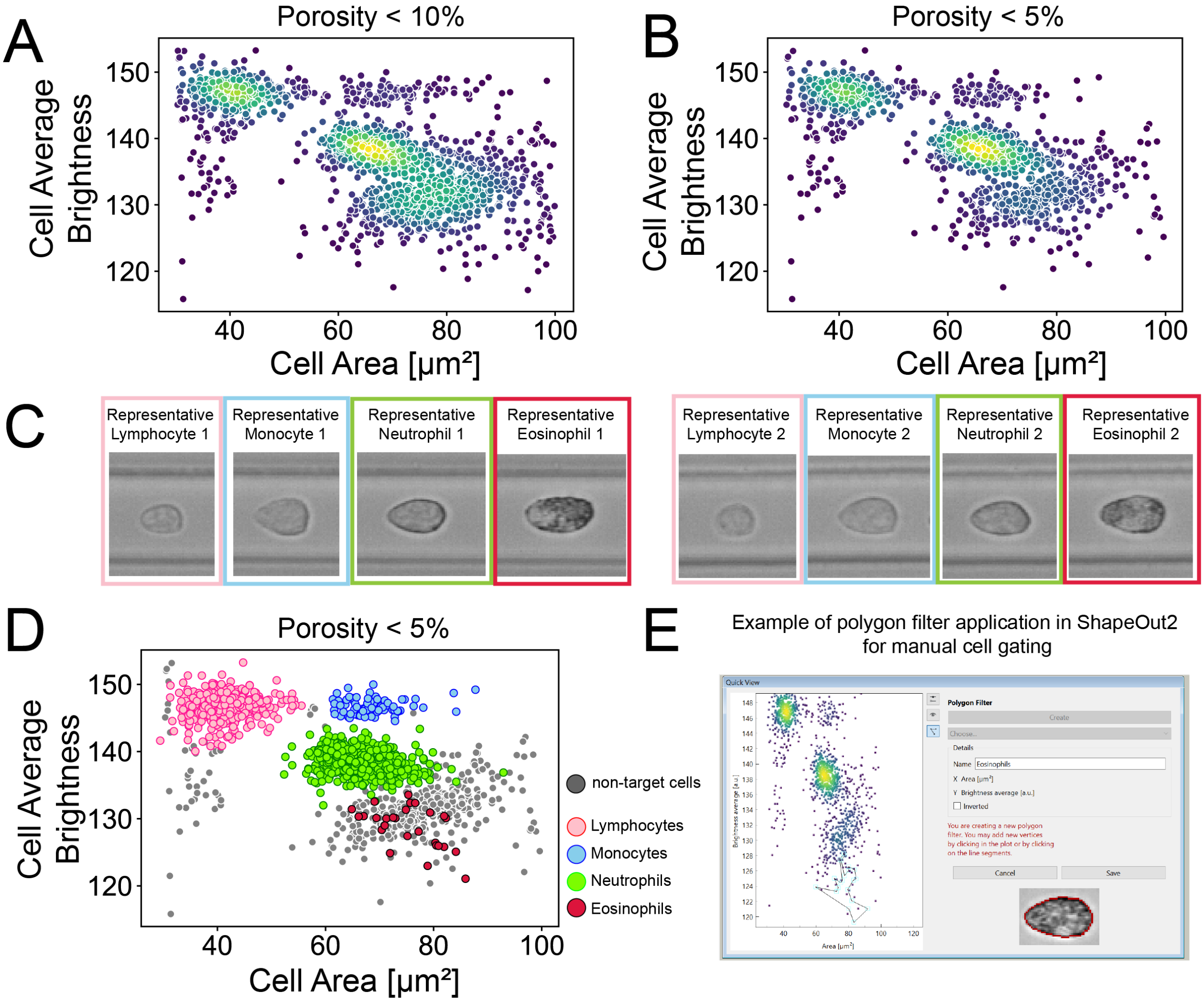

FIG. S3: Manual gating of WBCs and corresponding results. (A) Kernel density estimate (KDE) plot of cells selected using standard WBC box filters for cell area and deformation, with an additional porosity filter between 0% and 10%. (B) KDE plot from the same measurement applying a stricter porosity threshold of 5%. (C) Example images of the main WBC types in 20 μm microfluidic channel. (D) Final WBC populations identified through manual gating, overlaid on the KDE plot. (D) Example screenshot from ShapOut2 software showing manual gating of eosinophil population from surrounding RBC doublets based on visual inspection of cells.

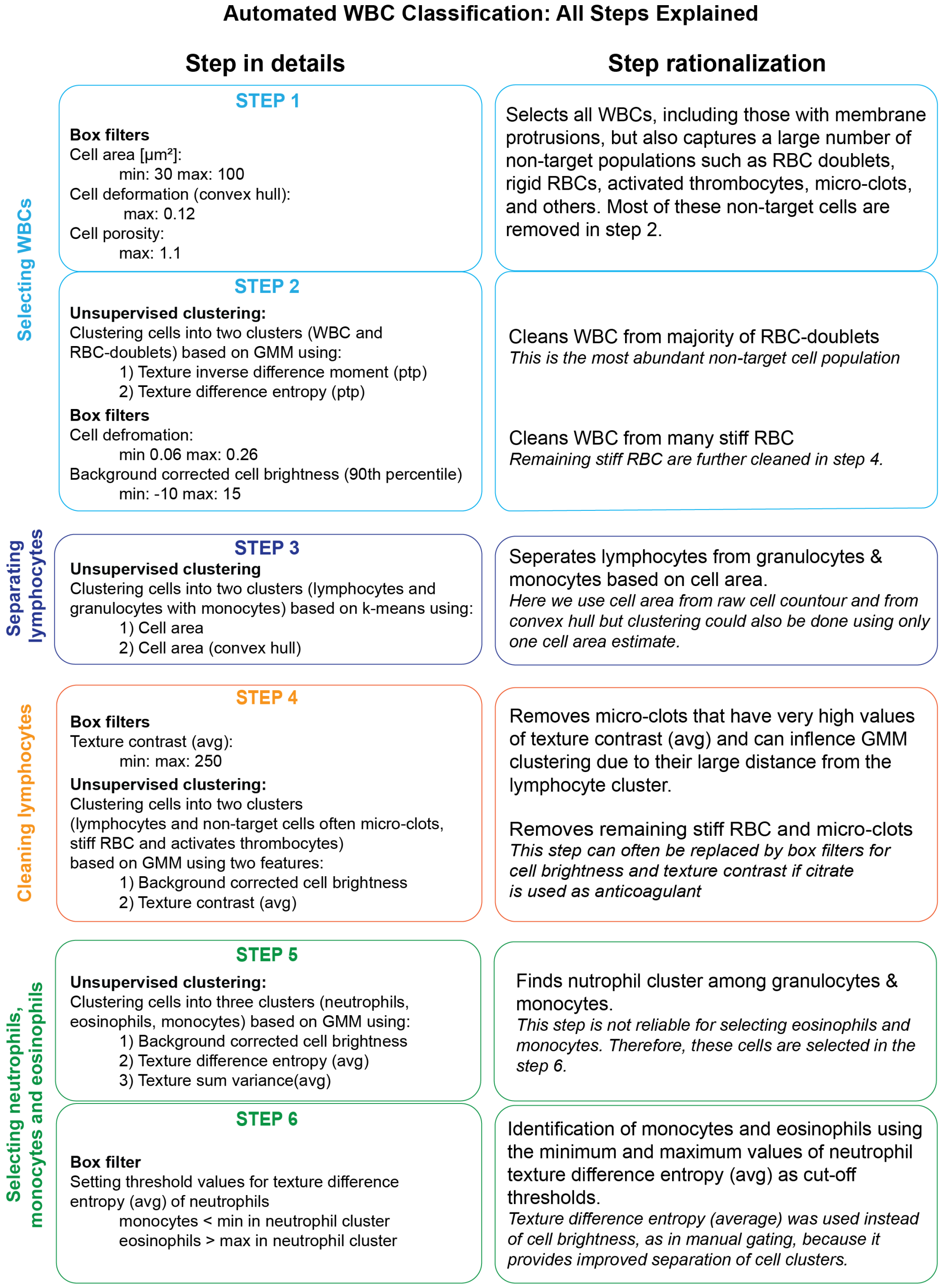

FIG. S4: Overview and rationale of the six-step automated WBC classification algorithm. These steps provide a general framework for classifying WBC types that can be adapted to different experimental conditions.

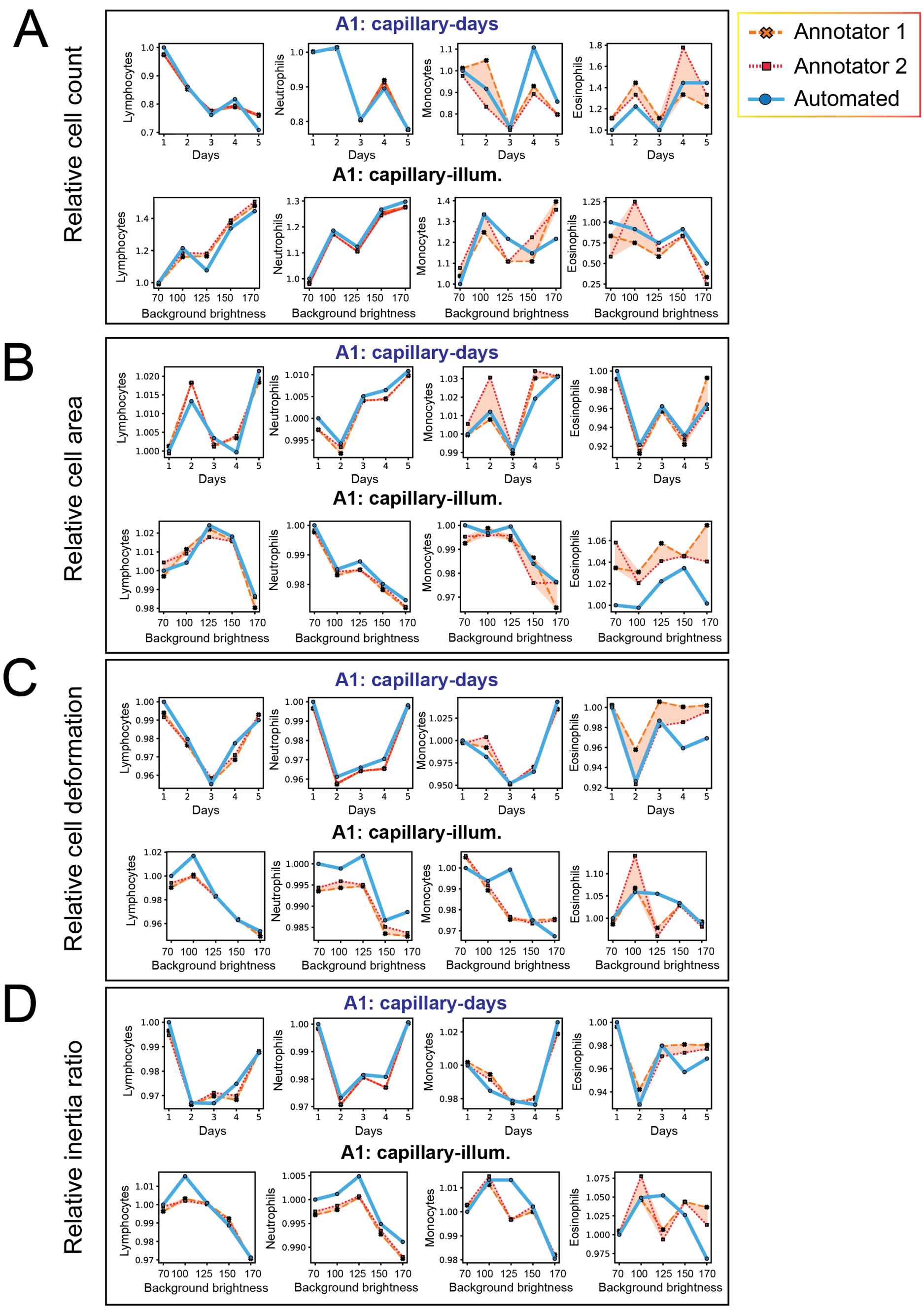

FIG. S5: Comparison of variation in cell counts and features between two human experts (manual gating) and automated classification. Two experimental sets were analyzed: (1) A1: capillary-days, where capillary blood was collected over five sequential days, and (2) A1: capillary-illum., where capillary blood was measured on the same day under varying background brightness (70–170 grayscale values). (A) WBC counts normalized to automated classification from the first measurement. (B) Cell area, (C) cell deformation, and (D) inertia ratio, each normalized to automated gating values from the first measurement in the respective experimental set.
