# Supplementary material for: Automation and improvement of WBC mechanical profiling in deformability cytometry
Source: Biophys J. 2025 Oct 10;125(12):3048–59. doi: 10.1016/j.bpj.2025.10.007 (PMC13507180; doi:10.1016/j.bpj.2025.10.007)
Supplement: Supplementary file 2 [file mmc3.pdf]

Biophysical Journal
Article

Automation and improvement of WBC mechanical profiling in deformability cytometry

Sara Kaliman,[1,2,*] Shada Abuhattum,[1,2] Benedikt Hartmann,[1,2,3] and Jochen Guck[1,2,3]
[1]Max-Planck-Zentrum für Physik und Medizin, 91054 Erlangen, Germany; [2]Max Planck Institute for the Science of Light, 91058 Erlangen, Germany; and [3]Friedrich-Alexander-Universität Erlangen-Nürnberg (FAU), 91054 Erlangen, Germany

ABSTRACT Deformability cytometry (DC) is a powerful biophysical technique that enables cost-effective, high-throughput characterization of disease-associated changes in blood cell mechanics. Mechanical profiling of living white blood cells (WBCs) is particularly valuable due to their critical role in the immune response. However, reliably identifying and classifying WBC subtypes in a label-free manner remains a significant challenge. Until now, the analysis pipeline has relied on manual gating by trained experts, limiting scalability and reproducibility. In this study, we present a fully automated and generalizable framework for WBC classification in shear flow DC experiments, based on box filters and unsupervised clustering of cell populations. Both box filters and unsupervised clustering rely on cell shape features derived from high-accuracy segmentation and on cell texture features derived from bright-field images. This unsupervised approach not only improves reproducibility and reduces processing time but also overcomes key limitations of supervised models that require extensive training data and often suffer from reduced performance under varying imaging conditions. We validated our method by comparing cell features obtained through manual gating and automated classification across six experimental sets. These sets incorporated variations in blood donors, anticoagulants (EDTA and citrate), blood collection sources (capillary and venous), and device brightness settings. Each set included five repeated measurements. The results consistently confirmed the reliability and robustness of the method across all tested conditions and WBC types. Importantly, this automated pipeline enables the inclusion of WBCs with membrane protrusions—typically excluded from standard analyses—allowing for morphological characterization of potentially activated cells. Moreover, by using shape features derived from the original contour rather than the convex hull, we improve morphological accuracy and reduce measurement variability. This approach thus enhances the accuracy, consistency, and scalability of WBC mechanophenotyping and enables high-throughput analysis across large cohorts.

SIGNIFICANCE To support the clinical adoption of deformability cytometry for white blood cell (WBC) mechanophenotyping, we present a scalable, accurate, and reproducible method for automated cell selection based on unsupervised clustering. This approach removes the need for manual gating and remains robust across anticoagulants, blood source, and illumination settings. Importantly, it includes WBCs with membrane protrusions, enabling future analysis of potentially activated cells. When combined with a small U-Net segmentation model, this methodology enables precise WBC mechanical profiling that can be promptly done even with a central processing unit (CPU). These improvements provide a reliable foundation for large-scale clinical studies of WBC mechanical properties, improving consistency, speed, and accuracy in research and diagnostic applications.

INTRODUCTION

Although the importance of cell mechanical properties in cell biological and biomedical contexts is now widely acknowledged, achieving reproducible and reliable mechanical characterization remains challenging—primarily due to variability in both experimental setups and data analysis approaches (1–3). However, recent advancements in machine learning offer promising opportunities to automate and standardize key aspects of data analysis. Ensuring reproducibility and accurately quantifying measurement errors are essential steps, particularly for clinical applications of high-throughput techniques such as deformability cytometry (DC) (4–7). In DC, single-cell mechanical properties are evaluated by subjecting cells to controlled deformation as they pass through a microfluidic channel. Various

Submitted May 30, 2025, and accepted for publication October 6, 2025.
*Correspondence: sara.kaliman@mpzpm.mpg.de
Dr. Jochen Guck passed away on October 3, 2025.
Editor: Joachim Raedler.
https://doi.org/10.1016/j.bpj.2025.10.007
© 2025 The Author(s). Published by Elsevier Inc. on behalf of Biophysical Society.
This is an open access article under the CC BY license (http://creativecommons.org/licenses/by/4.0/).

physical forces—including hydrodynamic, electrical, optical, and acoustic—can be used to induce this deformation (4). In this work, we focus on shear flow DC (sDC), which applies hydrodynamic shear stress through the controlled flow of cells through high-viscosity measurement buffers (MBs) in narrow channels (5,7). This established technique enables high-throughput mechanical characterization of cells. Shape features, such as cross-sectional area and deformation, are calculated from bright-field images recorded of up to several thousand cells per second. Traditionally, cell classification in sDC has relied exclusively on manual gating based on area and brightness metrics derived from these images (8).

Red blood cells (RBCs) are the most abundant cell type in blood, with approximately 600–700 single RBCs imaged for every white blood cell (WBC) in sDC measurements. Given this abundance, minor misclassifications of RBCs have little effect on overall population statistics. In contrast, accurate identification of WBCs is far more sensitive to contamination by nontarget cell types. Common sources of contamination include RBC doublets, less-deformed RBCs, micro-clots, and activated thrombocytes. These cell types often overlap with WBCs in terms of area, deformation, and brightness, making manual gating particularly difficult. Moreover, manual cell gating is time consuming and subject to user variability, limiting reproducibility and hindering large-scale studies that involve hundreds of patient samples.

Machine learning now offers promising tools to address long-standing challenges in cell classification for DC. Recent efforts have focused on convolutional neural networks, which take cropped bright-field images as input and, as output, assign to these images the probabilities for each defined cell class (9,10). Although this offers many advantages, including the ability to classify every recorded cell and cell agglomerate, these pretrained models are often sensitive to user-dependent settings, such as brightness and focus, leading to significant prediction errors when applied to datasets that differ from those they were originally trained on. Retraining these models typically requires large amounts of annotated data. Moreover, convolutional neural networks are large models and can suffer from long inference times when graphics processing units (GPUs) are unavailable—a common limitation in clinical environments.

In our previous work, we demonstrated that supervised machine learning can enable fast and accurate segmentation of cells recorded in a microfluidic channel. Here, we show that cell-feature-based unsupervised clustering can be used to classify WBCs with an accuracy comparable to manual gating by a human expert while improving reproducibility and efficiency. Given the central role of WBCs in the immune response and evidence that their mechanical properties change in various disease states (8,11,12), it is crucial to classify them in a rapid, flexible, and reliable manner. Moreover, the advances in segmentation from our previous work now enable the extraction of more detailed shape features, allowing for better morphological analysis.

Besides shape features, our unsupervised clustering utilizes texture features. When Haralick texture features (13) are used, cell populations can be separated better than when relying on cell brightness. Automated clustering of cell subpopulations can be done with algorithms, such as Gaussian mixture models (GMMs) (14) or *k*-means (15), applied to shape and texture cell features. We show that automated classification of WBCs yields results consistent with manual cell gating. This also allows for the inclusion of the entire WBC population—not only cells without membrane protrusions, as is often the case in manual cell gating. This is important because the ratio of WBCs with membrane protrusions in the entire population may serve as an indicator of immune system activation. Furthermore, we enhance WBC feature extraction by calculating shape features from the original cell contour rather than relying on the convex hull—a standard practice in sDC (7,8).

In addition, we demonstrate that our automated classification algorithm remains robust across a range of hematological and experimental conditions, including variations in donor age and gender, anticoagulant type, blood collection method, and illumination settings. We also quantify the variability of extracted features across repeated measurements. Together, the results of this work contribute to more consistent and transparent mechanical phenotyping, representing an important step toward standardized data analysis and a better understanding of how experimental factors influence WBC mechanics.

MATERIALS AND METHODS

Measurement protocol

Blood measurements were performed using both a custom-built and a commercial sDC device (AcCellerator, Zellmechanik Dresden, Dresden, Germany) as previously described (8), with minor modifications. A total of 10 μL of whole blood was mixed with 190 μL of MB (0.6% methyl cellulose in phosphate-buffered saline). This mixture was aspirated into sample tubing prefilled with MB using a syringe pump set to a reverse flow rate of -2 μL/s. The microfluidic chip, fabricated from polydimethylsiloxane, featured a constriction channel with a cross-sectional area of 20×20 μm^2 and a length of 500 μm. Cells were flowed through the channel at a total flow rate of 0.06 μL/s, composed of a sample flow of 0.015 μL/s and a sheath flow of 0.045 μL/s. Under these conditions, cells experienced hydrodynamic shear stresses sufficient to induce deformation. The region of interest, where cell images were recorded, was positioned near the end of the constriction channel, where steady-state deformation is achieved. Bright-field images (80×250 pixels) were acquired at a frame rate of 3600 Hz for a total measurement duration of 10 min. Cell segmentation was carried out using a small U-Net architecture designed and trained for sDC measurements (16).

Blood measurements

Numerous factors can influence the properties of blood cells. Although pathological changes due to disease are an obvious source of variation,

even blood from healthy donors may exhibit significant differences arising from biological variability, storage conditions, anticoagulant type, and the method of blood collection.

One common example of WBC mis-classification is the inclusion of RBC doublets and less-deformed RBCs, especially when using standard gating based on area, deformation, and porosity (see Figs. 1 *B* and S1). Our observations indicate that venous blood treated with EDTA (ethylenediaminetetraacetic acid) consistently contains more of these less-deformed RBCs and doublets compared to citrate-treated samples. In contrast, capillary blood collected without anticoagulants shows more variability in nontarget cell abundance (Fig. S1). Additionally, user-defined illumination settings on the sDC device significantly affect image brightness, thereby influencing key features, such as measured cell brightness and texture—both crucial for accurate WBC gating.

Our objective was to validate the performance of the automated WBC classification algorithm under a range of donor- and protocol-dependent conditions that commonly vary in real-world experimental settings. Additionally, we assessed the stability of cell features calculated from the original cell contour across repeated measurements within the same set of experiments.

To this end, we collected six experimental sets, each consisting of five repeated sDC measurements. We used blood from three donors. The first donor was a healthy adult male (A1) in his twenties, whereas the other two donors were pediatric patients (C1 and C2). Donor C1 was a 2-year-old female, and donor C2 was a 4-year-old male. Both C1 and C2 were oncology patients diagnosed with solid tumors rather than hematological malignancies. Informed consent was obtained from all donors. Ethical approval for donors C1 and C2 (application ID: 13_21 Bc, date: January

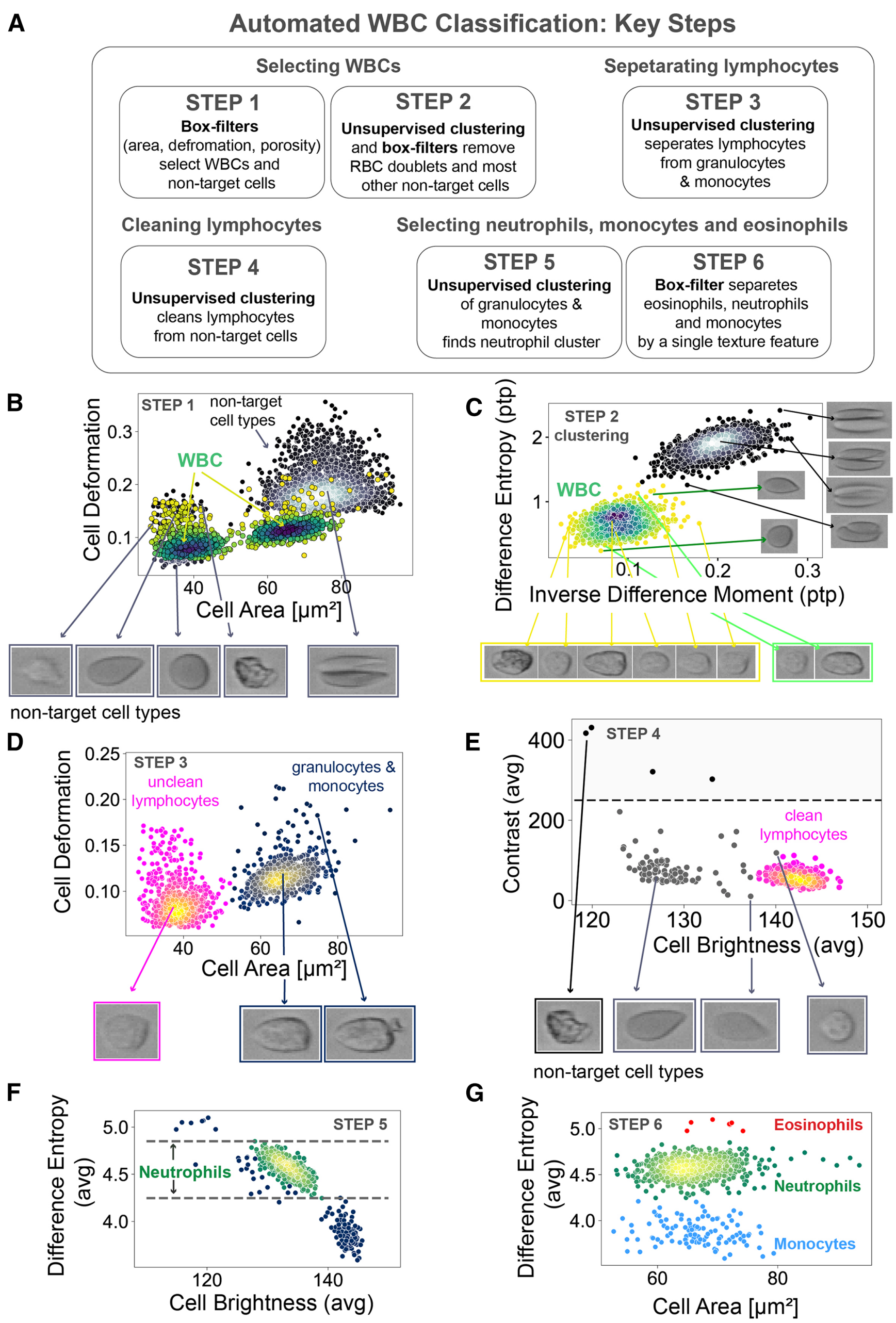

FIGURE 1 Schematic overview of the method for automated WBC classification. (*A*) Overview of the main steps used to automate WBC classification broken into six steps. (*B*) Initial WBC box filters (*step 1*) include many nontarget cell types. (*C*) Unsupervised clustering based on texture features (*step 2*) removes the majority of RBC doublets. (*D*) After removal of nontarget cells, the remaining WBCs are separated into two main clusters, namely, lymphocytes and granulocytes and monocytes, based on cell area (*step 3*). (*E*) Lymphocyte cluster is further refined to exclude remaining nontarget cells constituting mainly from micro-clots and less-deformed RBCs (*step 4*). (*F*) The granulocyte and monocyte group is clustered into three subpopulations using three features: avg cell brightness, avg difference entropy and avg sum variance. This step correctly identifies the neutrophil cluster and determines the minimum and maximum values of avg difference entropy for neutrophils (*step 5*). (*G*) Cells with avg difference entropy values exceeding the maximum observed for neutrophils are classified as eosinophils, whereas those with values below the minimum are classified as monocytes (*step 6*).

19, 2021) was granted by the Ethical Committee of the Faculty of Medicine, Friedrich-Alexander-University Erlangen-Nuremberg.

1) A1: venous-EDTA: venous blood from A1 was collected in an EDTA tube and measured five times within a 1.5-h period.
2) A1: venous-citrate: venous blood from A1 was collected in a citrate tube and measured five times within a 1.5-h period.
3) A1: capillary-days: capillary blood was collected from A1 and measured five times over the course of 1 week (once per day).
4) A1: capillary-illum.: capillary blood from A1 was measured at different illuminations. Blood was measured five times on the same day, with each sample collected from a different finger. In each sDC measurement, a different background brightness was set, namely, 70, 100, 125, 150, and 170 on the grayscale (0–255).
5) C1: venous-EDTA: venous blood from C1 was collected in EDTA and measured five times within a 1.5-h period.
6) C2: venous-EDTA: venous blood from C2 was collected in EDTA and measured five times within a 1.5-h period.

When venous blood was used, the tube was gently inverted before each sDC measurement. In experimental sets 1 and 2, the venous blood tubes were gently mixed on a roller mixer (10 rotations/min) at room temperature immediately after collection. In experimental sets 5 and 6, the tubes were kept at room temperature without mixing. For capillary blood samples, no anticoagulants were used; the blood was collected directly from the finger and pipetted into the MB immediately after collection. To assess the robustness of the method against device- and camera-specific variability, different generations of sDC devices were used for experiments 1–4 and 5–6.

Shape feature extraction

Cell segmentation was performed using a small U-Net architecture trained on pixel-wise annotated bright-field images from sDC measurements (16). This segmentation approach enabled a more accurate extraction of cell boundaries than the threshold-based approach commonly used for segmentation of sDC measurements. After segmentation, two main categories of cell features were extracted: shape based and texture based. Shape features included the projected cell area and cell deformation, each computed from both the original segmented contour and the convex hull. Shape-based features were then computed following the standard approach described in (7). Specifically, the cell area was determined as the zeroth-order image moment of the segmented cell. Deformation was defined as

$$\text{deformation} = 1 - \frac{2\sqrt{\pi A}}{P},$$

where A is the projected cell area and P is the perimeter. The cell inertia ratio was defined as $I = \sqrt{\frac{I_1}{I_2}}$, where I_1 and I_2 are the two principal moments of inertia along the principal axes of the body. Cell porosity is the ratio of the convex hull area and the original cell area, and it measures how enclosed the cell shape is. An apparent Young's modulus was extracted from a numerical-simulation-based lookup table using the convex hull cell area and deformation values (17).

Haralick texture feature extraction

To capture structural information beyond morphology, we also extracted Haralick texture features from the grayscale cell images (13). These features are derived from the gray-level co-occurrence matrix, which quantifies how frequently pairs of pixels with specific gray values occur at a defined spatial relationship within the image. Haralick originally proposed 14 texture measures that can be computed from the gray-level co-occurrence matrix in four angular directions, reported either as averaged (avg) values or as peak-to-peak (ptp) values, resulting in a total of 28 possible features. Many of these measures are strongly correlated. For automated WBC classification, our algorithm utilizes five selected features: inverse difference moment (ptp), difference entropy (ptp), contrast (avg), difference entropy (avg), and sum variance (avg). Formal definitions of these measures are provided in the original publication (13), and their sensitivity to factors such as gray-level quantization, resolution, and noise has been previously described (18,19). Whereas some features are interpretable—for example, contrast reflects local variations in gray levels, and entropy reflects the degree of randomness or disorder—others are less intuitive from their mathematical definitions. Importantly, ptp values primarily capture shape-related information, whereas avg values are more sensitive to a cell's internal texture. For further intuition on the five features used in this study—and how they help distinguish, for example, RBC doublets or WBCs with irregular texture patterns—see Fig. S2.

Haralick features were computed using the `mahotas` Python library (20) from background-corrected bright-field images, in which pixels outside the segmented cell contour were set to zero.

Both shape and texture features were calculated from sDC bright-field images with Python v.3.12 (Python Software Foundation, Wilmington, Delaware) using the custom-made software package `dcnum`, available at https://github.com/DC-analysis/dcnum.

Manual cell gating of WBCs

Manual gating of WBCs was performed according to the standard morpho-rheological analysis framework (8). Cells were filtered to include only those with porosity below 5%, and polygon filters were then applied in the cell area-brightness space using the open-source software `ShapeOut2` (https://shapeout2.readthedocs.io/en/stable). Compared to a porosity cutoff of 10% (Fig. S3 *A*), applying a stricter threshold of 5% (Fig. S3 *B*) removed many RBCs and RBC aggregates, thereby simplifying the manual gating process. Manual gating of subpopulations was performed using the cell area (to distinguish lymphocytes from monocytes) and cell brightness (to separate monocytes, neutrophils, and eosinophils). Thresholds were determined by an experienced annotator based on the typical morphology of each cell type (Fig. S3 *C*), as established in (8) based on magnetic cell sorting, controlled by fluorescence markers. This approach yielded distinct populations of neutrophils, monocytes, and eosinophils (Fig. S3 *D*). Notably, eosinophils often appear as dark as RBC doublets, which are far more abundant, and therefore require careful placement of polygon filters guided by cell images (Fig. S3 *E*).

However, this method also excluded WBCs with nonconvex shapes that exceeded the standard porosity cutoff of 5%. Additionally, manual gating is inherently subjective and prone to variability, especially for low-abundance subtypes such as eosinophils and monocytes, which may overlap in feature space with nontarget cells (Fig. S3 *D*).

Filtering and unsupervised clustering

Our algorithm for automated WBC classification uses six modular steps that rely on box filters and unsupervised clustering. Box filtering removes cells or cell aggregates with features outside a specified range. Measurement data are stored in HDF5 format, adhering to established metadata and cell feature standards. These files can be processed using Python packages such as `h5py`. Here, we utilized a custom-made Python package, `dclab` (https://github.com/DC-analysis/dclab, v.0.62.6), for analyzing sDC measurements and filtering cells based on their features. This box filtering can also be performed using the `ShapeOut2` graphical user interface (GUI), which is built on top of `dclab` and was used in the case of manual cell gating.

For unsupervised clustering, we used k-means (15) and GMMs (14), and we implemented both methods using the Python package `scikit-learn` (v.1.6.1). The k-means clustering method is based on Lloyd's algorithm and produces convex and isotropic clusters that minimize Euclidean distance to

the cluster centroids. On the other hand, GMM is a soft clustering method that assigns a probability that the data point belongs to a given cluster. It relies on the assumption that data points are generated from a mixture of Gaussian distributions with unknown parameters. Besides mean and covariance (spread of the function), these parameters also include the mixing probability or a weight for a given Gaussian. The GMM clustering method is well suited for elliptical clusters, whereas *k*-means is limited to circular cluster shapes (*k*-means can also be seen as a special case of GMM with equal covariance for each component).

We specified the number of clusters for both methods, using two or three clusters depending on the specific need of a given step. Cell features that can cluster the cells most effectively were carefully selected upon visual investigation of cells in the `ShapeOut2` software. For both the *k*-means and GMM algorithms, we specified the number of clusters and the random seed, whereas the other parameters were kept as the default. In `scikit-learn`, each component (in our case, the cell feature) has its own general covariance matrix by default.

RESULTS

Algorithm for automated WBC classification

Although RBCs can typically be identified using simple box filters that apply upper and lower thresholds to specific cell features, WBCs pose a more significant challenge. There are two reasons for this: one is the relatively low number of those cells and the other is the overlap in feature space with other cell types. Since even a small contamination from nontarget populations can change WBC feature values, more advanced classification strategies are required. Here, we present a detailed methodology for cleaning and classifying major WBC subtypes in whole blood. After the initial filtering to isolate WBCs from the overall cell population, WBC subtypes are refined and separated using box filters and unsupervised clustering. The whole classification algorithm can be structured into six distinct, modular steps (Fig. 1 *A*).

This modular approach can also be adapted from the analysis of blood from healthy adult human donors for neonatal blood, hematological disorders, nonhuman and blood (e.g., mouse blood), as well as for experiments involving changes in flow rate, MB composition, or device configuration. However, in hematological disorders that significantly alter WBC morphology, cell classification becomes challenging—not only for the automated approach presented here but also if manual gating is performed. Additionally, this framework provides a generalizable strategy for circumventing manual cell gating, with potential applicability across various types of DC.

Selecting WBCs

The first step of the algorithm applies broad inclusion filters to preliminarily isolate the WBC population. Cells are selected based on a convex hull area between 30 and 100 μm^2, a convex hull deformation of less than 0.12, and a porosity below 10%. These thresholds are designed to include all major WBC subtypes while excluding the majority of RBCs and cellular debris. Despite this, several nontarget populations pass the initial filters, including RBC doublets, less-deformed or lysed RBCs, micro-clots, and activated thrombocytes (Fig. 1 *B*).

In the second step, we removed the most abundant nontarget cell population—RBC doublets. Two ptp Haralick features—inverse difference moment and texture difference entropy—proved to be the most effective in this step, as these features are highly sensitive to local texture variation. RBC doublets, due to overlapping membranes, generate high-contrast textures that lead to elevated ptp values in both texture features (see Fig. S3). In contrast, single WBCs typically exhibit smoother, more uniform textures with lower local variability. By applying GMM clustering in this two-feature space, cells naturally segregated into two robust clusters: WBCs with low ptp values and RBC doublets with high values (Fig. 1 *C*). This step significantly improved the accuracy of WBC isolation and reduced the contamination by RBC aggregates early in the pipeline.

To remove the remaining nontarget populations, we applied a brightness-based filter using the 90th percentile of background-corrected cell intensity, which was found to be more robust than the average brightness. WBCs typically fall within a range of -10 to 15 grayscale units relative to the background (zero values). Less-deformed RBCs appear darker and fall below this lower threshold, whereas micro-clots are often overly bright and exceed the upper threshold. Finally, we utilized cell deformation calculated from the original cell contour and applied an additional filter for deformation between 0.06 and 0.26. This step improved the exclusion of residual aggregates and less-deformed RBCs.

Separating lymphocytes

After the initial filtering and removing most of the nontarget cells, in the third step, lymphocytes are separated from granulocytes and monocytes. The primary feature used for this separation was cell area, as lymphocytes are generally smaller than the other WBC types. To robustly distinguish these populations, we used the cell area calculated from both the original and convex hull contours and applied *k*-means clustering. This centroid-based approach is well suited for separating size-dependent populations, as it assigns cells to the nearest cluster center based on area (Fig. 1 *D*).

Cleaning the lymphocyte population

Although the initial *k*-means clustering effectively partitioned smaller lymphocytes from larger granulocytes and monocytes, the lymphocyte group may still contain residual nontarget cells—particularly less-deformed RBCs, very bright (possibly lysed) RBCs, micro-clots, and activated thrombocytes. This issue is especially pronounced for blood samples collected in EDTA tubes, which tend to contain a

higher proportion of less-deformed RBCs (see Fig. S2). To enhance lymphocyte purity, we applied an additional cleaning step using GMM clustering in the space of average cell brightness and average texture contrast (step 4). Before clustering, cells with texture contrast values exceeding 250 were excluded, as these outliers—primarily corresponding to highly contrasted micro-clots—could negatively affect the performance of unsupervised clustering (Fig. 1 *E*, *dashed line*). The remaining cells were clustered using GMM to identify and remove outliers. Less-deformed RBCs typically show similar contrast values to lymphocytes but much lower brightness, whereas micro-clots and activated thrombocytes exhibit higher texture contrast with brightness levels similar to lymphocytes. This two-step filtering process ensured a cleaner lymphocyte population. However, this step may require adaptation for specific blood samples. In some cases, no cleaning is needed, whereas in others, it is essential due to a high proportion of nontarget cells. The abundance and composition of these cells are strongly influenced by preanalytical variables, such as anticoagulant type, storage conditions, and time to measurement.

Neutrophil, monocyte, and eosinophil clustering

Granulocytes and monocytes are further subclustered into three major WBC types: neutrophils, monocytes, and eosinophils. In step 5, neutrophils are identified using GMM clustering in a three-dimensional feature space combining average cell brightness with two Haralick texture features—average difference entropy and average sum variance. Neutrophils, being the most abundant subtype, consistently form a well-defined cluster (Fig. 1 *F*). In contrast, monocytes and eosinophils are less abundant and show morphological variability, which often leads to less reliable identification in this step.

In the conventional morpho-rheological analysis framework, classification of neutrophils, monocytes, and eosinophils was performed manually, based on cell brightness (8). We investigated whether texture features could separate cell clusters more effectively than brightness alone. Several averaged texture features, including difference entropy, entropy, sum entropy, contrast, sum variance, variance, angular second moment, and inverse difference moment, showed promise for discrimination of the three cell types. Among these, average texture difference entropy provided the greatest separation between clusters (Fig. 1 *F*). Therefore, in step 6, monocytes and eosinophils were identified based on this feature. The cluster of cells that has an average difference entropy lower than the minimal value for the neutrophil cluster was classified as monocytes. On the contrary, we classify as eosinophils the cluster of cells with higher values of this texture feature than the maximum for the neutrophils (Fig. 1 *G*). Since classification is done based on texture and not cell brightness (see Video S1), a partial overlap in cell brightness can be expected. Basophils were excluded from this classification pipeline, as they constitute a very small fraction of circulating WBCs and do not consistently form separable clusters in the chosen feature space.

Adapting the algorithm

This modular workflow provides a general template that can be adapted by others employing imaging flow cytometry for the analysis of blood measurements. In sDC blood measurements, most of the detected events correspond to RBCs. Since we aim for WBCs, the first step is to filter out the majority of RBCs by applying box filters that isolate the region where WBCs are located. Once this region is defined, clustering algorithms are applied to group cells with similar features. For example, lymphocytes can be separated from granulocytes and monocytes based on their size. However, granulocyte and monocyte subtypes have the same area and deformation but different brightness and texture features (see Video S1). At multiple points throughout this process, each cluster may still contain nontarget cells, so additional cleaning steps are applied as needed rather than at a single stage.

We outline the algorithm in six steps (Fig. 1 *A*), with details and the rationale for each step described in Fig. S4. In addition, we have made the Python implementation of the algorithm publicly available. For convenience, each cell type is exported as a separate RTDC file. The algorithm executes in less than 1 min when storing only scalar features and in under 10 min when bright-field images are included. Before applying the algorithm, raw sDC data are preprocessed using U-Net-based segmentation (16) to obtain more reliable contours. In addition to shape descriptors, cell texture features are also computed.

An exploratory phase using visualization software, such as `ShapeOut2` for sDC data, is essential for identifying the most informative features and setting appropriate thresholds. This step assumes that the user is familiar with the morphology of the target cells; if not, fluorescent staining of the target cells should be performed beforehand. If, however, a human expert cannot distinguish the target cell types from the nontarget ones, based on their features or how they look, automation will also not be possible.

Feature-level validation of automated WBC classification against manual gating

To evaluate the performance of our automated WBC classification algorithm, we compared the extracted cell features with those obtained through manual gating—considered to be the gold standard—for each experimental set. This comparison was limited to WBCs with porosity below 5%, consistent with standard gating criteria. We assessed several conventional shape features: cell area and deformation (both computed from the convex hull), inertia ratio, and the apparent Young's modulus.

Across all experimental sets, cell feature values from our automated classification method closely matched those from manual gating. This agreement was especially strong for the most abundant WBC types, such as lymphocytes (typically 200–900 cells per measurement) and neutrophils (typically 200–1800 cells per measurement) (Fig. 2, *A* and *B*), demonstrating the reliability and robustness of the algorithm across different donors, anticoagulants, collection methods, and imaging conditions.

From our observations, users typically set the image brightness between 70 and 170 grayscale values. Therefore, an important test of our algorithm was an experimental set in which illumination settings were varied (A1: capillary-illum.). User-dependent adjustments of brightness and focus can affect not only the overall cell intensity but also texture-related features. This variability is one of the reasons why automated WBC classification must rely on unsupervised clustering rather than solely on box filters. Across five different illumination settings, our algorithm demonstrated as stable a performance as the manual gating, with no evidence of brightness-dependent data drift (see Fig. S5). In this experimental set, more deformed neutrophils were present, so the automated classifier yielded slightly higher deformation and inertia ratios than manual gating by including these cells without capturing nontargets.

Monocytes and eosinophils, being less abundant in typical 10-min sDC measurements (with often fewer than 150 and 10 cells, respectively), naturally exhibited greater feature variability. Nonetheless, in most cases, the automated method yielded consistent estimates that aligned well with manual gating (Fig. 2, *C* and *D*). Minor discrepancies between the two methods, such as the cell area (convex hull) of monocytes in donor C1, were observed occasionally, but these are likely attributable to the small sample sizes.

Importantly, a similar degree of variability was found when comparing manual gates created independently by two human experts using the same criteria (see Fig. S5). This supports the conclusion that much of the observed variability stems from the inherent sparsity of these rare cell populations. These findings highlight a broader issue in sDC: which magnitude of differences between groups of rare cell types can be interpreted as clinically important?

Impact of contour definition on within-measurement variability of cellular features

We argue that the nonconvex shapes frequently observed in WBCs reflect biological reality rather than segmentation errors. In the past, threshold-based segmentation methods have often introduced nonconvex contour artifacts, but using the convex hull of the contour provided more stable shape features. With the advent of more advanced segmentation techniques, we can now extract and analyze shape features directly from the original cell contour.

To demonstrate the benefits of this approach, we evaluated the stability of the WBC area and deformation by calculating the coefficient of variation (CV) across five repeated measurements for six experimental sets. In addition to supporting the reliability of nonconvex shape features, this evaluation was essential for quantifying within-sample variability arising from multiple sources and provides a general indication of the precision achievable in sDC measurements using advanced segmentation methods. To decouple errors due to feature extraction from the cell classification errors, WBCs were manually gated by an expert (convex hull/original area ratio of less than 5%).

Cell area derived from the original contour was very similar to the area obtained from the convex hull, indicating a very small discrepancy between the two methods. Furthermore, the CVs of the cell area were nearly identical for both representations (Fig. 3 *B*). Across all experiments, the CV remained below 2% for neutrophils and lymphocytes, the most abundant cell types, and reached up to 3% for monocytes.

In contrast, cell deformation values were significantly higher when calculated from the original cell contour compared to deformation from the convex hull of the contour. This increase results from the substantially larger perimeter associated with the nonconvex boundary (Fig. 3 *A*). However, the CV for deformation computed from the original contour was consistently lower than the deformation from the convex hull (Fig. 3 *C*). This suggests that the cell deformation calculated from the original cell contour remained stable across repeated measurements. For deformation derived from the convex hull, the CVs reached up to 9% for lymphocytes, whereas neutrophils and monocytes showed more consistent values, below 4% and 6%, respectively. In comparison, deformation calculated from the original contour exhibited reduced variability for all three cell types, with CVs remaining below 4%.

Eosinophils displayed substantially greater variability in shape features. When using the convex hull, CVs reached up to 6% for area and 15% for deformation. Applying the original contour reduced deformation variability to approximately 9%, yet it remained higher than that observed in other WBC types. These findings raise the question of whether the number of eosinophils captured in standard sDC measurements is sufficient for robust mechanical characterization. They also highlight the need for protocol refinements to improve the reliability of mechanical feature estimation for eosinophils, a clinically relevant cell type involved in various immune and autoimmune disorders.

WBC shape analysis: Full population versus subset without membrane protrusions

So far, the standard analysis protocol has considered only WBCs with porosity below 5% (see Fig. 4 *A*) and using the convex hull of the cell contour for estimating the cell

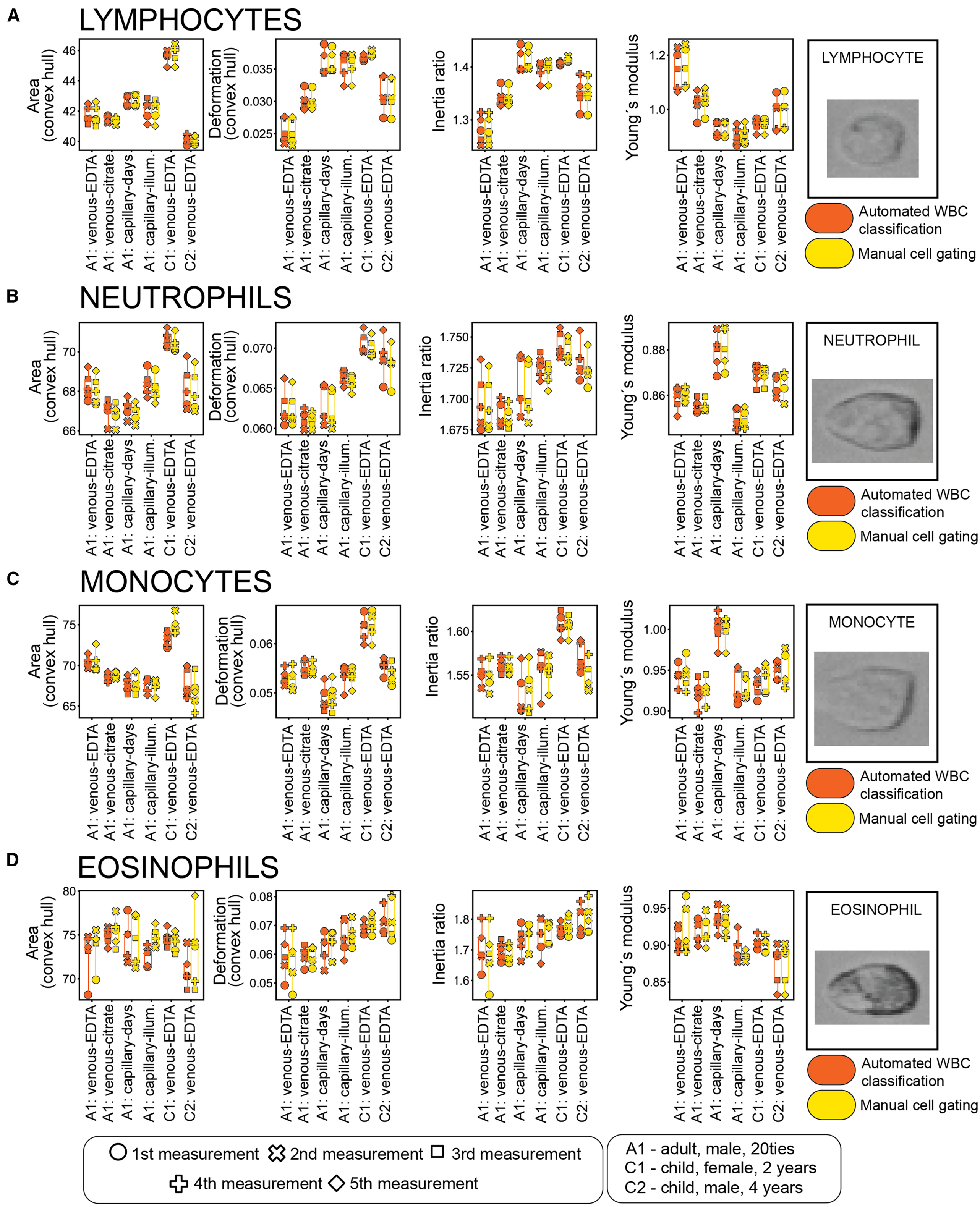

FIGURE 2 Comparison of cell features derived from manual gating versus automated WBC classification methods. Five sequential measurements were performed for each experimental set, which varied in donor identity, blood collection method (venous versus capillary), anticoagulant used (EDTA versus citrate), and measurement illumination settings. Mean values of the four most important cell features are compared between the two cell classification approaches for (*A*) lymphocytes, (*B*) neutrophils, (*C*) monocytes, and (*D*) eosinophils. Whiskers in the boxplot indicate the full range of the data. In all cases, differences in the calculated features between manual gating and automated WBC classification are substantially smaller than the variability observed across repeated measurements.

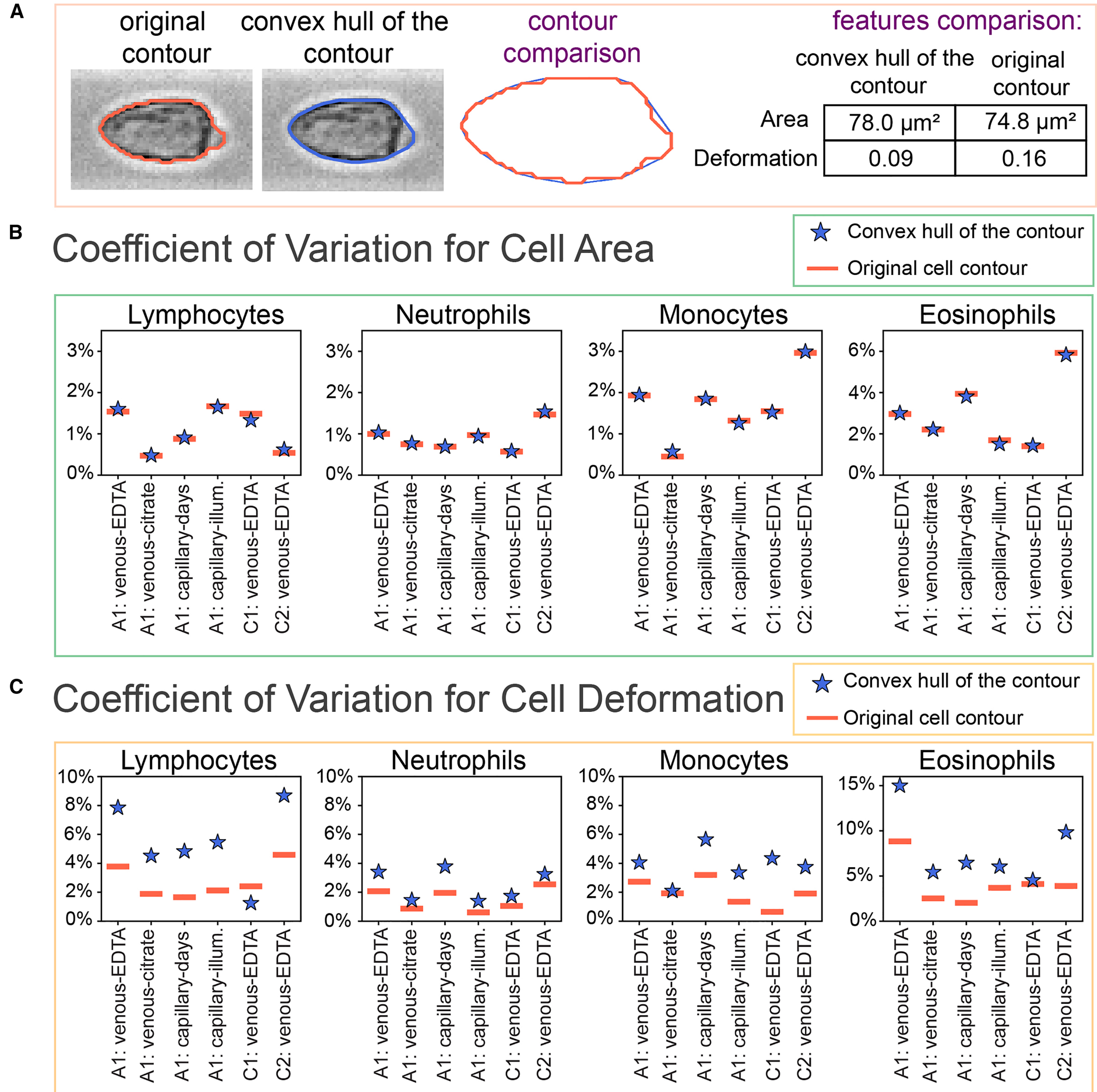

FIGURE 3 Comparison of variability in shape features derived from the original contours versus convex hulls in manually gated WBCs. (*A*) Example illustrating the difference in cell contours and corresponding feature values for a single neutrophil. (*B*) Coefficient of variation (CV) of the mean cell area across five repeated measurements and six experimental sets, comparing values obtained from the original contour and its convex hull. (*C*) CV of the mean cell deformation under the same conditions.

area and deformation. This ensures that membrane protrusions do not influence the calculation of mechanical features, as protrusions are not considered a result of bulk cell mechanics, such as stiffness, but rather a manifestation of local membrane curvature. Although the classical approach should still be used for estimating cellular mechanical properties, the new approach we are proposing here complements the classical one and contains additional information about the membrane protrusions that should be included in the analysis.

These membrane protrusions, seen in high-porosity cells (Fig. 4 *A*), may indicate WBC activation. Platelet-dependent granulocyte and monocyte activation is one known pathway (21,22) that can lead to the formation of protrusions and increased porosity. Recent studies have highlighted the importance of platelet-monocyte (23) and platelet-neutrophil

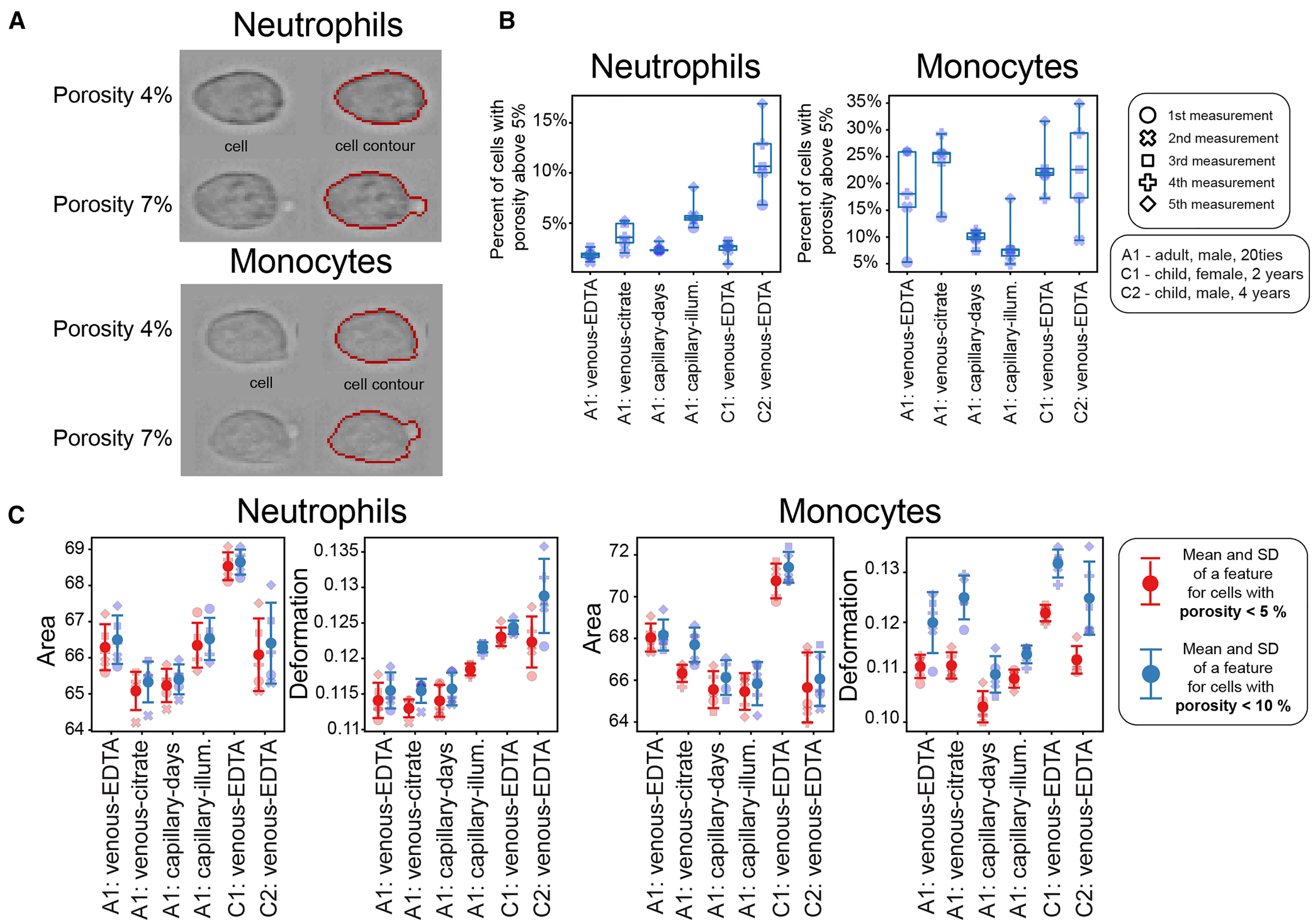

FIGURE 4 Analysis of WBCs exhibiting membrane protrusions. (*A*) Representative examples of segmented neutrophils and monocytes with low and high porosity; cells with high porosity display distinct membrane protrusions. (*B*) Proportion of high-porosity cells (porosity > 5%) within neutrophil and monocyte populations across five repeated measurements and six experimental sets. Whiskers in the boxplot indicate the full range of the data. (*C*) Comparison of cell area and deformation for neutrophils and monocytes calculated across the cell population subset without membrane protrusions (porosity < 5%) and the entire population (porosity < 10%). Error bars represent the mean and standard deviation across five repeated measurements for six experimental sets. The order of the repeated measurements in (*B*) and (*C*) is indicated by different symbols.

(24) interactions in disease. Therefore, including all WBCs in the classification and calculating the area and deformation from the original cell contour could provide additional information and reveal additional differences between patients.

We show that limiting the analysis to cells with porosity below 5% excludes up to 15% of neutrophils and 30% of monocytes (Fig. 4 *B*). Moreover, the percentage of high-porosity WBCs in a given blood sample could serve as an additional informative feature.

As expected, for low-porosity cells, the area was similar to the area of the whole population (porosity < 10%), whereas the cell deformation was lower. The extent of the difference in deformation between low-porosity subpopulations and whole neutrophil and monocyte populations depends on the fraction of high-porosity cells, as shown in Fig. 4, *B* and *C*. We also observed that differences between certain measurements became more pronounced when the entire WBC population was included in the analysis (Fig. 4 *C*).

DISCUSSION

The objective of this study was to enhance and automate WBC feature extraction in sDC experiments. A major obstacle to rapid feature extraction has been cell classification. We propose automating this step using cell-feature-based unsupervised clustering algorithms. This strategy enables efficient classification of major WBC types even on standard CPUs—commonly available in clinical settings. However, because we use feature-based clustering rather than image-based classification, this method must be applied after the measurement completion and not in real time. Thus, the advantage of real-time analysis in real-time DC (7), where the measurement duration can be flexibly adjusted when certain cell numbers are reached and cells can be sorted based on the morpho-rheological features detected (25,26), has to be traded for losing cells from the analysis or misclassifying them, which is especially pronounced in patient samples.

A critical component of this classification approach is the inclusion of Haralick texture features, which effectively distinguish WBCs from RBCs, characterized by smooth textures, as well as from micro-clots, which exhibit irregular textural patterns. This automatization paves the way for high-throughput analysis not only at the single-cell level but also across patient cohorts, supporting fast and reproducible assessments in large-scale clinical studies.

Although our results demonstrate robust performance across diverse blood samples, we acknowledge potential limitations under pathological conditions in which WBCs deviate significantly from typical clustering patterns. In such scenarios, a semiautomated approach, allowing for expert intervention via manual correction or initialization of cluster centroids, could provide a practical alternative. However, even expert assessments may be challenged by abnormal or ambiguous cell phenotypes. Notably, a failure of the algorithm to assign cells to expected WBC clusters may itself serve as a diagnostic cue, potentially signaling the presence of a hematological disorder or systemic disease.

In addition to automating classification, we propose improvements in feature extraction. Traditional sDC segmentation relied on thresholding, often leading to errors in identifying cell shapes. With recent advances in deep-learning-based segmentation, cell contour changes due to local membrane curvature can now be incorporated into the quantification of cell deformation. Such curvature perturbations may be biologically informative, potentially serving as indicators of WBC activation states. To leverage this, our classification algorithm includes WBCs with porosity—defined as the ratio of convex hull area to original cell area—up to 10%, a threshold that includes nearly all WBCs. We also introduce a new metric that may reflect immune activation: the percentage of WBCs in a sample with porosity greater than 5%.

Furthermore, we advocate using shape features such as cell area and deformation derived directly from the original cell contour, which better preserves morphological details in cells. These features show comparable or lower CVs across five repeated measurements compared to convex-hull-based features. This refined approach complements traditional mechanical metrics such as apparent Young's modulus, which is typically based on convex-hull-derived features for low-porosity cells, by providing deeper morphological insights potentially linked to cell activation.

Finally, when analyzing blood in different anticoagulants, we observed notable differences in the prevalence of less-deformed RBCs surrounding the lymphocyte population. Specifically, EDTA-treated samples consistently showed a greater number of small, round RBCs than citrate-treated ones. We also observed variations in WBC mechanical properties from the same donor depending on the anticoagulant used. These findings underscore the importance of further investigating preanalytical variables—such as anticoagulant type, collection method, and storage conditions—that can introduce hematological variability and potentially confound mechanophenotyping. Importantly, our results suggest that within-sample variability may, in some cases, be compared to interpatient differences, highlighting the necessity of robust error quantification to define meaningful thresholds for detecting disease-induced changes in WBC mechanics using sDC. Of note, our modular framework is not limited to the analysis of sDC measurements but is transferable and could facilitate automation in other cytometric platforms, for example, in flow cytometry, where manual gating remains a standard.

DATA AND CODE AVAILABILITY

Raw RTDC data files (HDF5 format) from six sDC experimental sets are publicly available at DCOR: https://dcor.mpl.mpg.de/organization/raw_data_wbc_classification. The bright-field images contained in these datasets were segmented using the small U-Net architecture (16), and cell features were subsequently extracted and added to the RTDC files with `dcnum`.

A Python implementation of the automated WBC classification algorithm is freely accessible at https://github.com/SaraKaliman/CytoClassify-WBC-classification-for-sDC.

ACKNOWLEDGMENTS

We thank Lena Summa and Felix Reichel for their contributions to the sDC experiments. Microfluidic chips used in this study were kindly provided by Parth Patel and Salvatore Girardo (Core Facility Lab-on-a-Chip, Max-Planck-Zentrum für Physik und Medizin). We are also grateful to Prof. Manfred Rauh, Prof. Markus Metzler, and Markéta Kubánková for their support in obtaining samples from the Pediatrics Department at University Hospital Erlangen. This project was supported by funding from Bayern Innovativ ("SoPhyA" - LSM-2303-0012), the Bayerische Staatsministerium für Gesundheit und Pflege ("disCOVer 2.0" - Gesamt-2490-PC-2023-V6), and core funding from the Max Planck Society. Finally, we dedicate this work to Prof. Erich Sackmann, whose pioneering contributions to biophysics have been a lasting source of inspiration and without whom this research would not have been possible.

AUTHOR CONTRIBUTIONS

Writing – original draft, S.K.; writing – review & editing, S.A., B.H., and J. G.; methodology, S.K.; conceptualization, S.K. and S.A.; validation, S.K.; formal analysis, S.K.; software, S.K.; investigation, S.K., S.A., and B.H.; visualization, S.K.; data curation, S.K. and S.A.; supervision, J.G.; funding acquisition, J.G.

DECLARATION OF INTERESTS

S.A. and J.G. are co-founders and shareholders of the company Rivercyte GmbH, which commercializes products for DC analysis of blood samples.

DECLARATION OF GENERATIVE AI AND AI-ASSISTED TECHNOLOGIES IN THE WRITING PROCESS

During the preparation of this work, the authors used ChatGPT with the GPT-4-turbo model to improve readability and language. After using this

tool, the authors reviewed and edited the content as needed and take full responsibility for the content of the publication.

SUPPORTING MATERIAL

Supporting material can be found online at https://doi.org/10.1016/j.bpj.2025.10.007.

REFERENCES

1. Pérez-Domínguez, S., S. G. Kulkarni, …, M. Lekka. 2023. Reliable, standardized measurements for cell mechanical properties. *Nanoscale.* 15:16371–16380.
2. Wu, P.-H., D. R. B. Aroush, …, D. Wirtz. 2018. A comparison of methods to assess cell mechanical properties. *Nat. Methods.* 15:491–498.
3. Yin, Y., and Z. Liu. 2024. Microfluidic Technology for Measuring Mechanical Properties of Single Cells and Its Application. *Bioengineering.* 11:1266. https://www.mdpi.com/2306-5354/11/12/1266.
4. Chen, Y., K. Guo, …, N. Xiang. 2023. Microfluidic deformability cytometry: A review. *Talanta.* 251:123815.
5. Urbanska, M., H. Muñoz, …, J. Guck. 2020. A comparison of microfluidic methods for high-throughput cell deformability measurements. *Nat. Methods.* 17:1–7.
6. Fajdiga, L., Š. Zemljič, …, J. Derganc. 2025. Shear flow deformability cytometry: A microfluidic method advancing towards clinical use - A review. *Anal. Chim. Acta.* 1355:343894. https://www.sciencedirect.com/science/article/pii/S0003267025002880.
7. Otto, O., P. Rosendahl, …, J. Guck. 2015. Real-time deformability cytometry: On-the-fly cell mechanical phenotyping. *Nat. Methods.* 12:199–202.
8. Toepfner, N., C. Herold, …, J. Guck. 2018. Detection of human disease conditions by single-cell morpho-rheological phenotyping of blood. *eLife.* 7:e29213. https://doi.org/10.7554/eLife.29213.
9. Kräter, M., S. Abuhattum, …, M. Herbig. 2021. AIDeveloper: deep learning image classification in life science and beyond. *Adv. Sci.* 8:2003743.
10. Schuchardt, J. P., M. Kräter, …, W. S. Harris. 2024. Omega-3 supplementation changes the physical properties of leukocytes but not erythrocytes in healthy individuals: An exploratory trial. *Prostaglandins Leukot. Essent. Fatty Acids.* 202:102636. https://www.sciencedirect.com/science/article/pii/S0952327824000309.
11. Kubankova, M., B. Hohberger, …, M. Kraeter. 2021. Physical phenotype of blood cells is altered in COVID-19. *Biophys. J.* 120:2838–2847.
12. Crawford, K., A. DeWitt, …, H. O'Neal. 2018. Rapid Biophysical Analysis of Host Immune Cell Variations Associated with Sepsis. *Am. J. Respir. Crit. Care Med.* 198:280–282.
13. Haralick, R. M., K. Shanmugam, and I. Dinstein. 1973. Textural Features for Image Classification. *IEEE Trans. Syst. Man Cybern.* SMC-3:610–621.
14. Wolfe, J. H. 1970. Pattern Clustering by Multivariate Mixture Analysis. *Multivariate Behav. Res.* 5:329–350.
15. Lloyd, S. 1982. Least squares quantization in PCM. *IEEE Trans. Inf. Theor.* 28:129–137.
16. Kaliman, S., R. Alajangi, …, S. Abuhattum. 2025. Small U-Net for Fast and Reliable Segmentation in Imaging Flow Cytometry. *Cytometry. A.* 107:450–463.
17. Wittwer, L. D., F. Reichel, …, S. Aland. 2023. A new hyperelastic lookup table for RT-DC. *Soft Matter.* 19:2064–2073. https://doi.org/10.1039/D2SM01418A.
18. Brynolfsson, P., D. Nilsson, …, A. Garpebring. 2017. Haralick texture features from apparent diffusion coefficient (ADC) MRI images depend on imaging and pre-processing parameters. *Sci. Rep.* 7:4041.
19. Löfstedt, T., P. Brynolfsson, …, A. Garpebring. 2019. Gray-level invariant Haralick texture features. *PLoS One.* 14:e0212110.
20. Coelho, L. P. 2013. Mahotas: open source software for scriptable computer vision. *J. Open Res. Softw.* 1:e3.
21. Rinder, H. M., J. L. Bonan, …, B. R. Smith. 1991. Dynamics of leukocyte-platelet adhesion in whole blood. *Blood.* 78:1730–1737. https://doi.org/10.1182/blood.v78.7.1730.1730.
22. Peshkova, A. D., S. M. Saliakhutdinova, …, J. W. Weisel. 2024. The differential formation and composition of leukocyte-platelet aggregates induced by various cellular stimulants. *Thromb. Res.* 241:109092.
23. Fu, G., M. Deng, …, M. J. Scott. 2021. Platelet-Monocyte Aggregates: Understanding Mechanisms and Functions in Sepsis. *Shock.* 55:156–166.
24. Kaiser, R., R. Escaig, …, L. Nicolai. 2021. Neutrophil-Platelet Interactions as Novel Treatment Targets in Cardiovascular Disease. *Front. Cardiovasc. Med.* 8:824112. https://doi.org/10.3389/fcvm.2021.824112.
25. Nawaz, A. A., M. Urbanska, …, J. Guck. 2020. Intelligent image-based deformation-assisted cell sorting with molecular specificity. *Nat. Methods.* 17:595–599.
26. Nawaz, A. A., D. Soteriou, …, S. Girardo. 2023. Image-based cell sorting using focused travelling surface acoustic waves. *Lab Chip.* 23:372–387. https://doi.org/10.1039/D2LC00636G.

Biophysical Journal, Volume *125*

Supplemental information

Automation and improvement of WBC mechanical profiling in deformability cytometry

Sara Kaliman, Shada Abuhattum, Benedikt Hartmann, and Jochen Guck

Supplemental Information

Automation and Improvement of WBC Mechanical Profiling in Deformability Cytometry

Sara Kaliman,[1,2,*] Shada Abuhattum,[1,2] Benedikt Hartmann,[1,2,3] and Jochen Guck[1,2,3,†]

[1] *Max Planck Institute for the Science of Light, 91058 Erlangen, Germany*

[2] *Max-Planck-Zentrum für Physik und Medizin, 91058 Erlangen, Germany*

[3] *Friedrich-Alexander-Universität Erlangen-Nürnberg (FAU), 91054 Erlangen, Germany*

[*] sara.kaliman@mpl.mpg.de

[†] jochen.guck@mpl.mpg.de

I. SUPPLEMENTARY FIGURES

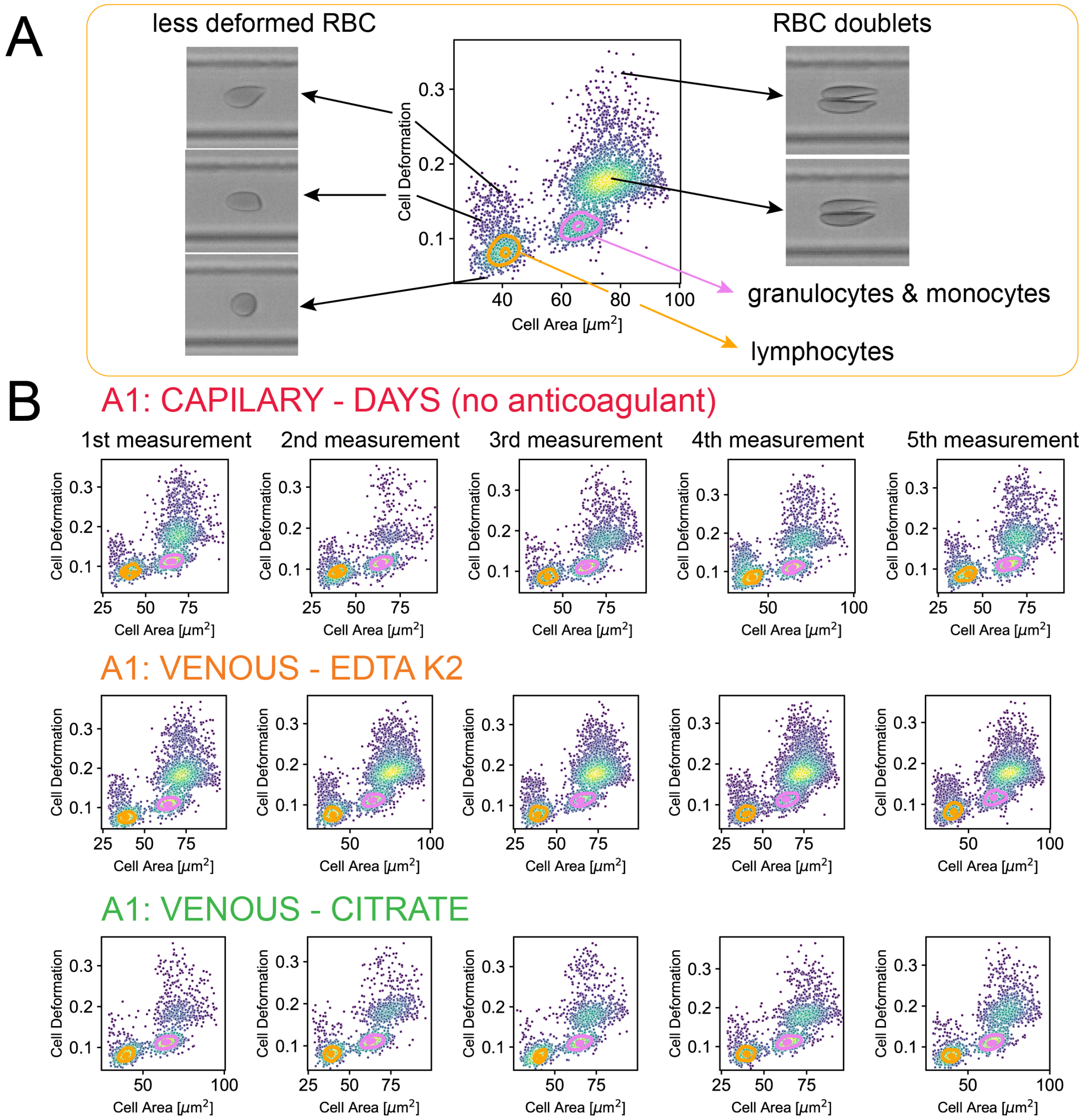

FIG. S1: Kernel density estimate (KDE) plot of target and non-target cell populations within standard WBC box-filters for area and deformation, and with porosity threshold of 10%. (A) Lymphocyte regions are predominantly surrounded by less deformed RBCs, whereas granulocytes and monocytes cluster is bordered by RBC doublets. (B) Comparison of non-target population abundance across three experimental sets, each comprising five measurements: A1: capillary-days (no anticoagulant was used), A1: venous-EDTA and A1: venous-citrate. All blood samples are collected from the same donor (A1) and KDE two levels indicate 50% and 95% of distribution density. Notably, using citrate results in fewer RBC doublets compared to using EDTA K2.

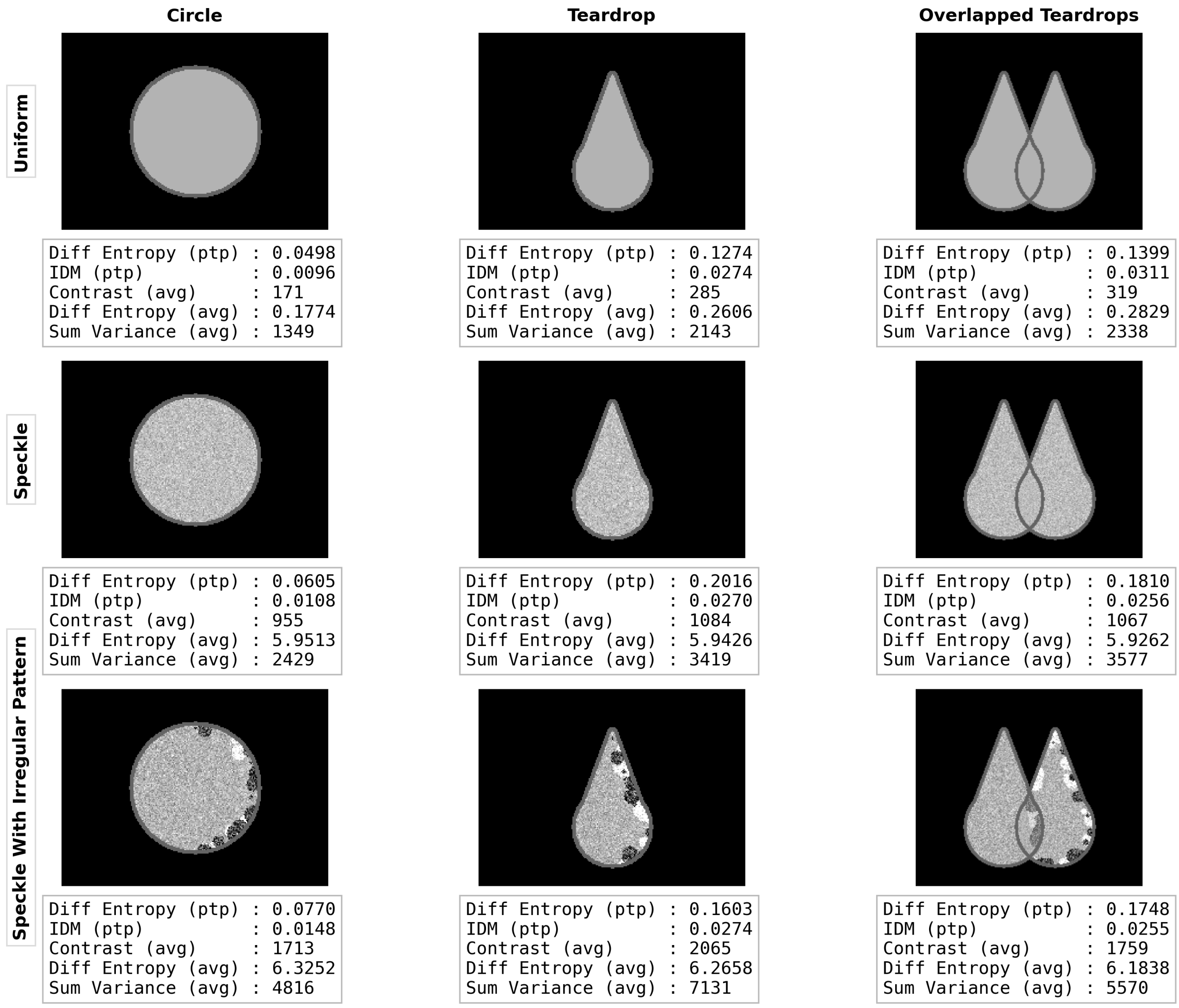

FIG. S2: Visual guide illustrating the five Haralick features used in automated WBC classification. Columns present synthetic shapes: circle, teardrop, overlapping teardrops, while rows depict different textures: uniform, speckle, and speckle with irregular patterns. These schematic renderings mimic the diversity of shapes and textures observed in cells captured by the initial box filters, including RBC doublets and WBCs. Among WBCs, some exhibit more regular speckled textures (e.g., lymphocytes and monocytes), while others display more irregular textures (e.g., eosinophils). For each image, the accompanying table reports values of: Difference Entropy (ptp), Inverse Difference Moment (IDM) (ptp), Contrast (avg), Difference Entropy (avg), and Sum Variance (avg)

.

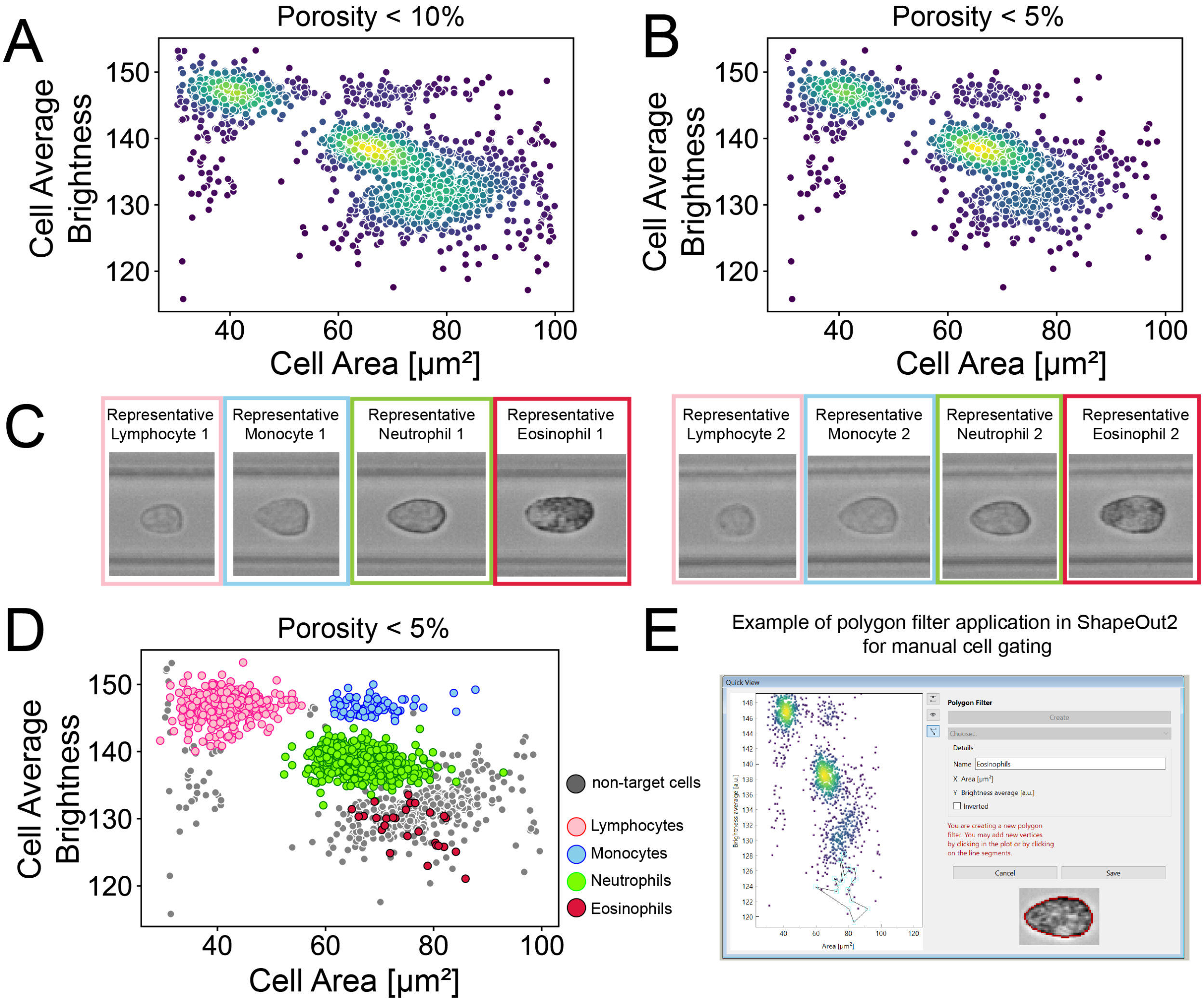

FIG. S3: Manual gating of WBCs and corresponding results. (A) Kernel density estimate (KDE) plot of cells selected using standard WBC box filters for cell area and deformation, with an additional porosity filter between 0% and 10%. (B) KDE plot from the same measurement applying a stricter porosity threshold of 5%. (C) Example images of the main WBC types in 20 μm microfluidic channel. (D) Final WBC populations identified through manual gating, overlaid on the KDE plot. (D) Example screenshot from ShapOut2 software showing manual gating of eosinophil population from surrounding RBC doublets based on visual inspection of cells.

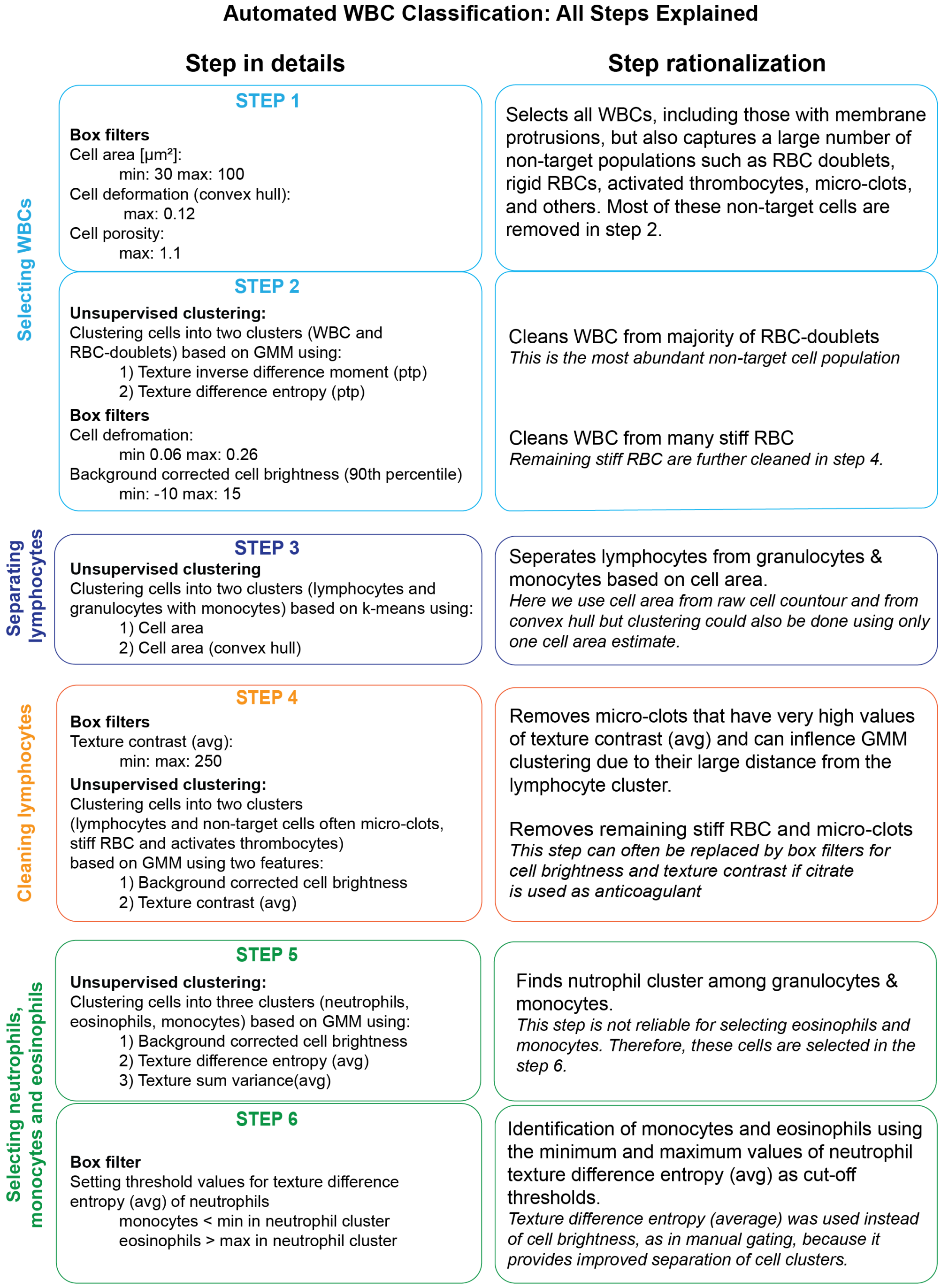

FIG. S4: Overview and rationale of the six-step automated WBC classification algorithm. These steps provide a general framework for classifying WBC types that can be adapted to different experimental conditions.

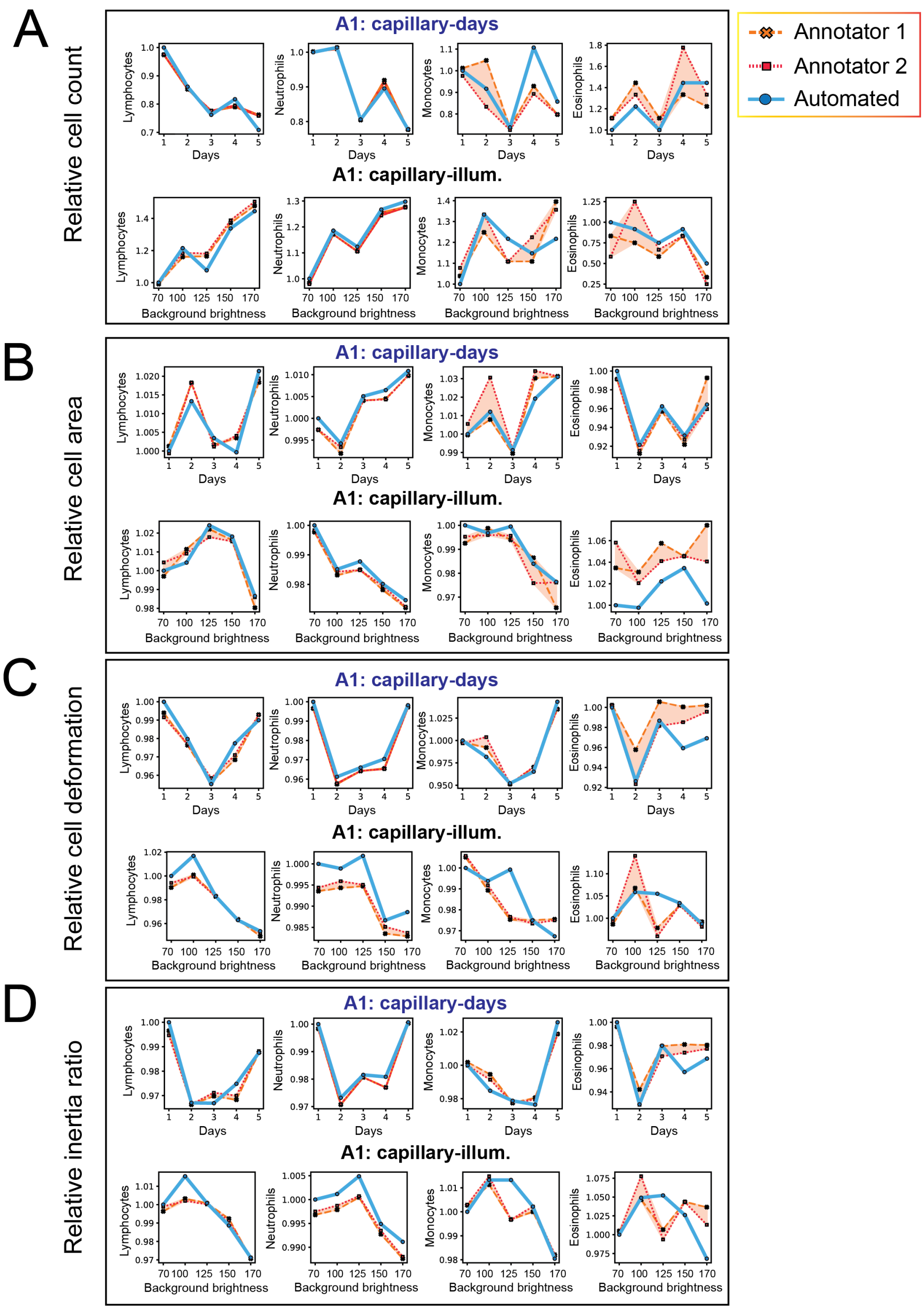

FIG. S5: Comparison of variation in cell counts and features between two human experts (manual gating) and automated classification. Two experimental sets were analyzed: (1) A1: capillary-days, where capillary blood was collected over five sequential days, and (2) A1: capillary-illum., where capillary blood was measured on the same day under varying background brightness (70–170 grayscale values). (A) WBC counts normalized to automated classification from the first measurement. (B) Cell area, (C) cell deformation, and (D) inertia ratio, each normalized to automated gating values from the first measurement in the respective experimental set.
